# Epidemiology and outcomes of pediatric dog bites: a retrospective cohort study at a tertiary pediatric emergency department

**DOI:** 10.1097/MS9.0000000000005742

**Published:** 2026-10-14

**Authors:** Nicholas W. Nakasian, Llwyatt K. Hofer, Charles A. Elmaraghy, Jill A. Foster, David L. Rogers

**Affiliations:** a Department of Ophthalmology, Nationwide Children’s Hospital, Columbus, OH, USA; b Ohio University Heritage College of Osteopathic Medicine, Dublin, OH, USA; c The University of Toledo College of Medicine and Life Sciences, Toledo, OH, USA; d Department of Otolaryngology, Nationwide Children’s Hospital, Columbus, OH, USA; e Department of Otolaryngology – Head and Neck Surgery, The Ohio State University, Columbus, OH, USA; f Department of Ophthalmology, The Ohio State University, Columbus, OH, USA

**Keywords:** cohort study, dog bite injuries, emergency department, pediatric trauma

## Abstract

**Background:**

Dog bites are a common cause of pediatric emergency department visits and represent a growing public health concern. This study aimed to describe the epidemiology, injury characteristics, bite circumstances, and clinical management of pediatric dog bite injuries.

**Materials and methods:**

We performed a retrospective chart review of consecutive patients younger than 18 years presenting to a tertiary pediatric emergency department for initial management of dog bites from January 2023 to December 2024. Patients evaluated more than 48 hours after their initial injury or with wounds initially treated at an outside institution were excluded. Outcomes of interest included patient demographics, injury characteristics, bite circumstances, and management strategies.

**Results:**

A total of 785 encounters were reviewed, with 711 encounters included; the mean age was 7.5 years, and 54% were male. Craniofacial injuries occurred in 63% of cases; these patients were younger, shorter, and lighter than those with only torso/extremity injuries (all *P* < 0.001). Operating room repair was required in 85 cases (12%), and 95 (13%) were hospitalized, with higher admission rates among patients with craniofacial injuries (17 vs 6.5%, *P* < 0.001). Oral antibiotics were prescribed for 692 patients (97%), and post-treatment wound infection occurred in 4% of patients without pre-existing infection. Bites occurred most frequently in spring and summer. Among cases with breed data (*n* = 372), pit bulls (26%) and mixed breeds (22%) were most common. Family dogs accounted for 41% of bites. Among cases with documented preceding activity (*n* = 441), 43% followed inappropriate child-dog interactions.

**Conclusion:**

Pediatric dog-bite injuries disproportionately affect younger children, commonly involve the craniofacial region, and often require advanced care. Most bites involve potentially preventable interactions, highlighting opportunities for targeted prevention and caregiver education.

## Introduction

Dog bite injuries are a common and preventable cause of pediatric emergency department (ED) visits and represent a significant public health concern. According to national data from the CDC’s Web-based Injury Statistics Query and Reporting System, dog bites ranked as the country’s eighth-leading cause of nonfatal injuries among children aged 5–9 years in 2023^[^1^]^. Multiple studies have reported a substantial increase in pediatric dog bite injuries in the United States during the COVID-19 pandemic, with higher dog ownership, adoption rates, and increased time spent at home as possible contributing factors^[^2,3^]^. Although the initial surge occurred during the pandemic, there is evidence that EDs have continued to see elevated numbers of dog bite injuries following the removal of lockdown measures compared with pre-pandemic levels. For example, a study from a large trauma center in the United Kingdom reported a 47% increase in dog bite presentations in the year following the end of lockdown measures (2022) compared with the year preceding the pandemic (2018)^[^4^]^. The increasing burden of pediatric dog bite injuries highlights the need for contemporary descriptive studies in this population.

HIGHLIGHTS

- This cohort contained 711 pediatric patients with dog bite injuries who presented to a tertiary care emergency department over a 2-year period.
- Injuries to the craniofacial region were most common (63%) and occurred in younger and smaller children.
- Craniofacial injuries were associated with higher hospitalization rates (17 vs. 6.5%).
- Dog bites occur more frequently during warmer months, and 46 unique breeds were indicated.
- Most bites involved familiar dogs and followed potentially preventable child-dog interactions.

Evidence from pre-pandemic studies shows pediatric dog bites most commonly occurred during the summer months, involved preschool-aged (1–5 years) and school-aged children (5–9 years), and were more likely to result in facial injuries in younger children^[^5–7^].^ Few studies, however, provide information regarding the circumstances of bite events and details of clinical management. Understanding patterns in child-dog interactions that lead to bite events is an essential step in optimizing dog-bite prevention education for families with young children. Additionally, detailed data on clinical management can help quantify the burden these injuries have on the health care system and further justify prevention efforts. To contribute to the available data in these categories, this study aimed to describe pediatric dog-bite injuries that presented to a large tertiary pediatric department in 2023 and 2024, with a focus on epidemiology, injury characteristics, and clinical management. By leveraging a large, contemporary cohort, we sought to better define post-pandemic pediatric dog-bite injury epidemiology and clinical management to optimize future prevention efforts and clinical care. This retrospective cohort study has been reported in accordance with the STROCSS 2025 guidelines^[^8^]^.

## Methods

This study was approved by the institutional review board at our institution on 24 April 2025 (MOD00025538). This study was conducted in accordance with the Declaration of Helsinki. A retrospective chart review was performed of all patients under the age of 18 who presented to the ED at a tertiary pediatric hospital for the initial evaluation and management of a dog bite injury between 1 January 2023 and 31 December 2024. Patients evaluated more than 48 hours after their initial injury were excluded due to the difference in treatment protocol for non-acute dog bite injuries. Patients referred from outside institutions were included if the initial wound management occurred at our institution. When the same patient presented to the ED for separate dog bite injuries during the study period, each visit was treated as an independent encounter and analyzed separately. Each bite event was considered a clinically distinct injury with its own circumstances, management, and outcome.

Data were collected from the electronic medical record and entered into a standardized database created using Research Electronic Data Capture (REDCap). Sources of data included ED provider notes, nursing/triage/procedural notes, imaging reports, and relevant follow-up visits. Data collected for patient demographics included age, height, weight, race/ethnicity, and sex. Data collected relevant to the circumstances of the dog bite incident included date of injury, dog breed, the child’s familiarity with the dog, and the activity leading to the bite. Any dog described as a combination of breeds or a “breed-mix” was categorized as a “mixed breed.” Descriptive data on treatment and outcomes included bite location, need for imaging, need for prophylactic antimicrobials, need for OR repair or hospitalization, and any infections at an injury site within 2 months of treatment. Anatomical injury categories were not mutually exclusive, as patients with injuries involving multiple sites were included in each applicable category. For comparisons between injury groups, patients were classified as having a craniofacial injury if they sustained at least one injury to the face, head, or neck, regardless of concomitant torso or extremity injuries. Patients were classified as non-craniofacial only if their injuries were limited to the torso or extremities. Patient outcomes were tracked for all subsequent encounters related to the initial dog bite injury. Missing data were excluded from variable-specific analyses and were reported as unknown.

Statistical analyses were primarily descriptive. Categorical variables, including patient demographics, dog characteristics, and details of the bite incident and injury management, were summarized as counts and percentages for comparison. Comparison of hospitalization rates between patients with craniofacial injuries and non-craniofacial injuries was performed using a chi-square test. Continuous variables such as patient age, height, and weight were summarized as means with standard deviations and compared between craniofacial and non-craniofacial injury groups using independent-samples *t*-tests. To evaluate whether craniofacial injury was independently associated with hospitalization, a multivariable binomial logistic regression was performed with hospitalization as the outcome and craniofacial injury, age, and weight as covariates. Weight was selected as the measure of body size rather than height to avoid collinearity between the two. The model was fit using forced entry on complete cases, where age, weight, and outcome were all present (*n* = 696). Adjusted odds ratios were reported with 95% confidence intervals. Because 7 of 711 encounters (~1%) represented visits by the same patient, a sensitivity analysis restricting the cohort to each patient’s first encounter (*n* = 704) was performed, repeating the primary chi-square and independent-samples *t*-test comparisons to confirm that the non-independence did not affect the findings. All statistical analyses were performed using JASP version 0.98.1 (JASP Team, University of Amsterdam), with statistical significance defined as a two-sided *P* < 0.05.

## Results

There were a total of 785 encounters presenting to our ED for management of dog bite injuries during 2023 and 2024. After excluding patients who presented more than 48 hours after injury and patients who had initial injury management at an outside institution, 711 encounters were included (Fig. 1). Seven patients presented to the ED with multiple bite injuries within the time frame of this study, and data were collected for each encounter. Patient demographics are summarized in Table 1, demonstrating a cohort with a slight male predominance, diverse racial representation, and a mean age of 7.5 years. Injuries most commonly involved the craniofacial region, and these patients were significantly younger (mean difference of 3.27 years, 95% CI: 2.62–3.92), shorter (mean difference of 21.94 cm, 95% CI: 17.71–26.17), and lighter (mean difference of 16.52 kg, 95% CI: 13.0–20.04; all *P* < 0.001) than those with injuries limited to the torso or extremities (Table 2). Patients with craniofacial injuries were also significantly more likely to require hospitalization than those with injuries limited to the torso or extremities [79/466, 17% (95% CI: 13.8–20.6%) vs 16/245, 6.5% (95% CI: 4.1–10.3%); *P* < 0.001]. In a sensitivity analysis restricted to first encounters (*n* = 704), all comparisons were materially unchanged, including the younger age, shorter height, and lower weight of the craniofacial group (all *P* < 0.001) and its higher hospitalization rate [77/462, 16.7% (95% CI: 13.5–20.3%) vs 16/242, 6.6% (95% CI: 4.1–10.5%); *P* < 0.001]. In a multivariable logistic regression of the full cohort, adjusted for age and weight, craniofacial injury remained independently associated with hospitalization (adjusted OR 2.56, 95% CI: 1.71–3.83, *P* < 0.001). Neither age (adjusted OR 0.93, 95% CI: 0.86–1.01) nor weight (adjusted OR 1.00, 95% CI: 0.99–1.02) was independently associated with hospitalization.

**Figure 1.**
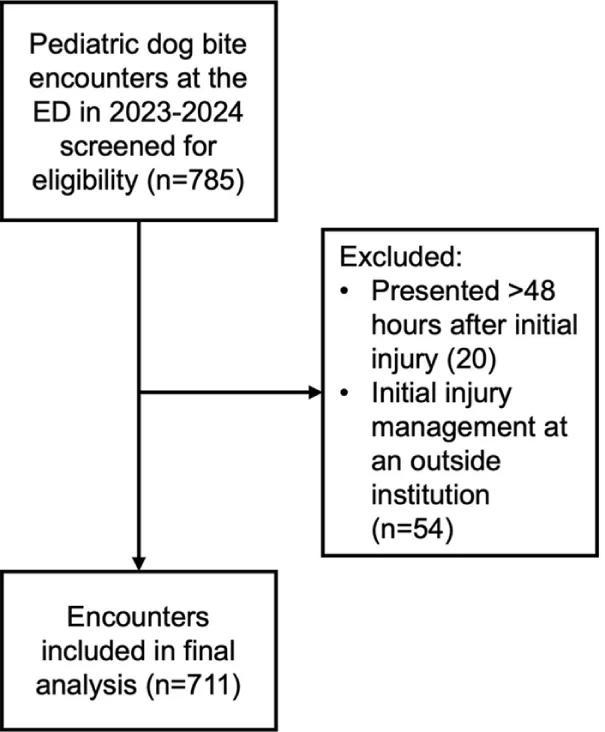
Selection of pediatric emergency department encounters for dog-bite injuries included in the study cohort.

Flow diagram depicting the selection of pediatric patients who presented to the emergency department (ED) with dog bite injuries. Of 785 encounters assessed for eligibility, 74 were excluded based on pre-specified exclusion criteria, resulting in 711 encounters being included in the final analysis.

**Table 1:** Patient demographics for 711 pediatric dog bite encounters at a single tertiary emergency department in 2023 and 2024.

Characteristic	Total (*N* = 711)
Sex	
Male	387 (54%)
Age (years)	
Mean ± SD	7.5 ± 4.4
Range	0.1–17.9
Age categories	
0–3 years	137 (19%)
3–6 years	164 (23%)
6–9 years	155 (22%)
9–12 years	128 (18%)
12–15 years	81 (11%)
15–18 years	46 (6%)
Race/ethnicity	
White	421 (59%)
Black	165 (23%)
Hispanic	40 (6%)
Asian	8 (1%)
Other^a^	77 (11%)
Height (cm)	
Mean ± SD	124.9 ± 29.8
Range	16.6–192.2
Weight (kg)	
Mean ± SD	33.6 ± 22.4
Range	5.6–141.4

^a^Patients listed as bi-racial.

**Table 2:** Age, height, and weight stratified by injury location for pediatric dog bites in the emergency department.

Characteristic	Face	Head/neck	Upper extremity	Lower extremity	Torso	Craniofacial (face + head/neck)	Non-craniofacial	*P*-value†
Total patients	451	55	171	97	43	466	245	
Age, years	6.43 ± 4.11	5.78 ± 4.29	9.55 ± 4.47	9.96 ± 3.78	8.71 ± 3.72	6.39 ± 4.10	9.66 ± 4.28	<0.001
Height, cm	117.64 ± 28.50	107.87 ± 28.0	138.63 ± 26.40	140.35 ± 23.86	132.71 ± 27.73	117.13 ± 28.66	139.07 ± 26.58	<0.001
Weight, kg	28.11 ± 19.26	26.53 ± 20.43	44.13 ± 25.09	45.77 ± 23.01	39.26 ± 25.29	27.97 ± 19.02	44.49 ± 24.38	<0.001

^a^ *P*-value compares craniofacial vs non-craniofacial groups.

Anatomical injury categories are not mutually exclusive; patients with injuries involving multiple sites were included in each applicable category. The craniofacial group includes patients with at least one face, head, or neck injury, regardless of concomitant torso or extremity injury. The non-craniofacial group includes patients whose injuries were limited to the torso and/or extremities.

Wounds were commonly treated with simple cleaning and closure in the ED, but additional care was often required, and specialist involvement was necessary for more complex injuries. Nearly all patients received antibiotic therapy, whereas fewer required imaging, operative repair, or hospitalization (Table 3). Fourteen patients arrived in the ED with pre-existing infections of their bite wounds. For those without a pre-existing infection, only 4% returned for follow-up treatment for a wound infection within 2 months of their injury. No patients in this cohort sustained fatal injuries.

**Table 3:** Additional interventions required for 711 pediatric dog bite patients presenting to the emergency department.

Treatment	Patients	Proportion
Imaging	154	22%
Oral ABX	692	97%
Topical ABX	477	67%
IV/IM ABX	78	11%
Rabies Vx	45	6%
OR repair	85	12%
Hospitalization	95	13%

ABX, antibiotics; IV, intravenous; IM, intramuscular; Vx, vaccine; OR, operating room

Bite events were most common during warmer months, with 35 and 26% of these cases taking place in the summer and spring, respectively. Of the 372 cases for which breed information was available, pit bulls were the most commonly reported biters (26%), followed by mixed breeds (22%), German shepherds (9%), huskies (6%), and Labrador retrievers (5%). A total of 46 unique breeds were involved. Breed data were unavailable in 48% of cases. Patients were often familiar with the offending dog, with family dogs being the most common biters (41%), followed by neighbors’ dogs (19%), relatives’ dogs (16%), friends’ dogs (11%), and stray dogs or those with an unfamiliar owner (10%). Data on dog familiarity were available in 691 of 711 cases (97%). In 441 cases, activities preceding the bite were described (Table 4). Most bite events followed inappropriate child–dog interactions, with fewer occurring during unprovoked or appropriate interactions.

**Table 4:** Type of interaction preceding dog bite event in 441 cases.

Interaction category	*n* (%)
Inappropriate interaction with dog	**187 (42)**
Provocation with dog’s food, toy, or treat	54 (12)
Rough play with dog	53 (12)
Trying to pet or play with unknown dog	31 (7)
Provocation of sleeping dog	29 (7)
Separating fighting dogs	20 (5)
Unprovoked bite	**102 (23)**
Unprovoked at home	51 (12)
Unprovoked away from home	51 (12)
Appropriate interaction with dog	**93 (21)**
Normal play with dog	56 (13)
Petting, cuddling, or hugging dog	37 (8)
Other interactions or accidental bite	**59 (13)**
Accidental provocation (tripping or falling onto dog)	21 (5)
Walking near unknown dog	20 (5)
Bike, scooter, or vehicle use near dog	12 (3)
Entering another home	6 (1)

Percentages are rounded to the nearest whole number.

## Discussion

Pediatric dog bites remain a significant public health concern. This study represents the largest single-center cohort of pediatric dog bite injuries since the lifting of COVID-19 lockdown protocols. Injuries most commonly involved the face and affected children younger than 9 years of age. Patients with craniofacial injuries were significantly younger, shorter, and lighter than those with injuries limited to the torso or extremities. While most cases were managed in the ED, a notable proportion required operative repair or hospitalization. Wound infection following treatment at our institution was uncommon. Our study findings reinforce previously established trends, including age-related vulnerability and craniofacial involvement, while providing contemporary data on injury patterns and bite circumstances in a pediatric population following the COVID-19 pandemic.

The increased prevalence of craniofacial injuries in younger, smaller children is likely multifactorial. Anatomically, a shorter child’s face is closer to the dog, increasing exposure. Developmentally, younger children may not be able to properly recognize signs of an animal’s distress and disengage from unsafe interactions. These factors likely explain the higher risk of craniofacial injuries in this group, consistent with prior pediatric dog bite studies^[^5,9–12^].^ Craniofacial injuries were also associated with greater injury severity, as reflected by higher hospitalization rates in our cohort. Prior studies similarly report increased operative intervention for head and neck injuries, with higher rates of deep tissue involvement, fractures, and injuries to critical structures that necessitate specialized surgical management and inpatient monitoring^[^6,11,13,14^]^.

Clinical management of pediatric dog bite injuries in this cohort demonstrated that most of these injuries can be effectively treated in the ED with simple wound care and closure. Nearly all patients received oral antibiotic prophylaxis. A minority of patients required operative repair (12.0%) or hospital admission (13.4%). These findings suggest that most pediatric dog bite injuries can be managed by the ED team and do not require operative repair or specialist intervention. Emergency providers must be able to properly assess injury severity to determine which patients can be treated in the ED and which require additional surgery or specialty services.

Recognition of common dog bite event patterns is essential for informing prevention strategies and education efforts. A higher frequency of bites during summer months is an established seasonal trend^[^7,11,12^],^ which likely reflects increased child–dog interactions or decreased adult supervision while children are out of school. The importance of supervising children at home, even with familiar animals, is highlighted by the high frequency of bite injuries from family-owned dogs. A total of 46 breeds caused injury in this study, so caregivers should recognize that bite injury risk extends beyond the breeds typically deemed more “aggressive,” such as pit bulls. Additionally, many bites resulted from inappropriate or modifiable interactions, suggesting that many of these injuries may be preventable with proper supervision and child education. Prevention strategies should focus on teaching safe child–dog interactions, consistent supervision, and increased awareness during warmer months. Pediatricians should be aware of the risks associated with dog-bite injuries and be able to properly inform families with children and dogs.

Many studies on pediatric dog-bite injuries provide robust data on patient demographics, anatomic sites of injury, and injury management, but offer limited detail on the circumstances of the event – such as dog breed, the activity leading to the bite, or whether the child was being supervised. We attempted to address this gap by manually reviewing ED visit notes for all identifiable, modifiable risk factors. Even with this intensive approach, activity preceding the bite was documented in only 62% of encounters, and breed information was absent in 48% of encounters. This variability and incompleteness in documentation highlight the need for standardized, comprehensive dog-bite templates in EDs to improve risk assessment and inform future prevention efforts.

As a single-center study in a tertiary pediatric ED, the findings may not be generalizable to all settings or populations. Its retrospective design limits the ability to establish causality and relies on the completeness of medical record documentation. Missing data were substantial for several variables, particularly dog breed, which was unavailable in 48% of cases (339/711), and preceding activity, which was unavailable in 38% of cases (270/711). These variables depend largely on caregiver reports and were not consistently documented. Therefore, the reported breed distributions and preceding activity patterns reflect only cases with available documentation and may not be representative of the full cohort. Missing data may introduce bias if the cases with documented information differed systematically from those without complete documentation. Inherently, certain details of bite events are difficult to capture, given that many incidents are unwitnessed and children may have limited ability to recall or accurately describe the interaction. Standardized documentation of dog bite encounters in the ED could improve both patient care and the quality of future research, facilitating comprehensive analyses of modifiable risk factors and circumstances contributing to these injuries.

Although conducted at a single tertiary pediatric center, the injury patterns observed in this study align with those reported in broader pediatric dog-bite literature. Additionally, our center serves as a regional referral center for severe pediatric injuries across our state and surrounding states, suggesting that our study population is broadly representative of the Midwestern United States. Pattern characteristics, injury locations, seasonality, involvement of familiar dogs, and diversity of implicated breeds are consistent with existing multicenter and population-based studies. These findings suggest that the trends identified are not unique to a single institution and may be applicable in similar clinical settings.

This cohort represents the largest single-center study of pediatric dog bite injuries since the discontinuation of COVID-19 lockdown protocols. Dog bite injuries in this cohort disproportionately affected younger children, commonly involved the craniofacial region, and often required advanced care. The data from this study indicate that pediatric dog bites often involve potentially preventable interactions, highlighting the opportunity for targeted prevention and caregiver education.

## Data Availability

The data that support the findings of this study are available from the corresponding author upon reasonable request, subject to institutional approval and applicable patient privacy and confidentiality requirements.

## Presentation

Preliminary data from this study were presented at the Ohio University Health Scholars Research Symposium (2025) and the Abigail Wexner Research Institute Annual Research Retreat (2025).

## References

[1] WISQARS leading causes of nonfatal injury visualization tool. Accessed 15 December 2025. https://wisqars.cdc.gov/lcnf/?y1=2023&y2=2023&ct=10&cc=0&s=0&g=00&a=lcd1age&a1=0&a2=199&d=0

[2] Menk PT Mahabee-Gittens EM Ruest S. Pediatric dog bite injury trends during the COVID-19 pandemic. Pediatr Emerg Care 2025;41:509. 10.1097/PEC.0000000000003378 40116052

[3] Menk PT Mahabee-Gittens EM Pomerantz WJ. The effects of the COVID-19 pandemic on pediatric dog bite injuries. Inj Epidemiol 2024;11:55. 10.1186/s40621-024-00537-x PMC11447959 39363346

[4] O’Hara N. Dog bites are increasing in frequency and severity - a sustained effect following the COVID-19 pandemic. J Plast Reconstr Aesthet Surg 2024;95:21–23. 10.1016/j.bjps.2024.05.049 38865840

[5] Patterson KN Horvath KZ Minneci PC. Pediatric dog bite injuries in the USA: a systematic review. World J Pediatr Surg 2022;5:e000281. 10.1136/wjps-2021-000281 PMC9716788 36474513

[6] McLoughlin RJ Cournoyer L Hirsh MP. Hospitalizations for pediatric dog bite injuries in the United States. J Pediatr Surg 2020;55:1228–33. 10.1016/j.jpedsurg.2019.06.025 31326111

[7] Kaye AE Belz JM Kirschner RE. Pediatric dog bite injuries: a 5-year review of the experience at the children’s hospital of philadelphia. Plastic Reconstr Surg 2009;124:551. 10.1097/PRS.0b013e3181addad9 19644273

[8] Agha RA Mathew G Rashid R. Revised strengthening the reporting of cohort, cross-sectional and case-control studies in surgery (STROCSS) guideline: an update for the age of Artificial Intelligence. PJS 2025;10:100081.

[9] Zangari A Cerigioni E Nino F. Dog bite injuries in a tertiary care children’s hospital: a seven-year review. Pediatr Int 2021;63:575–80. 10.1111/ped.14484 32979010

[10] Sulaiman A Liang D Gianoutsos M. Paediatric dog bite injuries: a 10-year retrospective cohort analysis from sydney children’s hospital. ANZ J Surg 2022;92:1149–52. 10.1111/ans.17581 35229428

[11] Plana NM Kalmar CL Cheung L. Pediatric dog bite injuries: a 5-year nationwide study and implications of the COVID-19 pandemic. J Craniofac Surg 2022;33:1436–40. 10.1097/SCS.0000000000008670 PMC9275798 35758430

[12] Loder RT. The demographics of dog bites in the United States. Heliyon 2019;5:e01360. 10.1016/j.heliyon.2019.e01360 PMC6431755 30957043

[13] Khan K Horswell BB Samanta D. Dog-bite injuries to the craniofacial region: an epidemiologic and pattern-of-injury review at a level 1 trauma center. J Oral Maxillofac Surg 2020;78:401–13. 10.1016/j.joms.2019.11.002 31816277

[14] Lee C Tiourin E Schuljak S. Surgical treatment of pediatric dog-bite wounds: a 5-year retrospective review. WestJEM 2021;22:1301–10. 10.5811/westjem.2021.9.52235 PMC8597704 34787555

